# Can Apple and Google continue as health app gatekeepers as well as distributors and developers?

**DOI:** 10.1038/s41746-023-00754-6

**Published:** 2023-01-31

**Authors:** Olamide Sadare, Tom Melvin, Hugh Harvey, Erik Vollebregt, Stephen Gilbert

**Affiliations:** 1 Else Kröner Fresenius Center for Digital Health, Technische Universität Dresden, Dresden, Germany; 2 School of Medicine, Trinity College, University of Dublin, Dublin, Ireland; 3 Hardian Health, Haywards Heath, England; 4 Axon Lawyers, Amsterdam, The Netherlands

**Keywords:** Health policy, Medical ethics

## Abstract

Mobile apps are the primary means by which consumers access digital health and wellness software, with delivery dominated by the ‘Apple App Store’ and the ‘Google Play Store’. Through these virtual storefronts Apple and Google act as the distributor (and sometimes, importer) of many thousands of health and wellness apps into the EU, some of which have a medical purpose. As a result of changes to EU law which came into effect in May 2021, they must now ensure that apps are compliant with medical devices regulation and to inform authorities of serious incidents arising from their use. The extent to which these new rules are being complied with in practice is uneven, and in some areas unclear. In light of EU legislation related to competition, which came into effect in November 2022, it is also unclear how conflicts of interest can be managed between Apple and Google’s roles as gateway duopoly importers and distributors whilst also developers of their own competitive health products. Finally, with the proposed European health data space regulation, wellness apps will be voluntarily registered and labelled in a fashion more like medical devices than consumer software. We explore the implications of these new regulations and propose future models that could resolve the apparent conflicts. All stakeholders would benefit from improved app store models to sustainably evolve safer, better, and fairer provision of digital health applications in the EU. As EU legislation comes into force it could serve as a template for other regions globally.

## Introduction

Apple and Google dominate the provision of apps through the ‘Apple App Store’ and the ‘Google Play Store’ respectively, accounting for around 92% of the mobile app distribution business^1^. The success of their vertically integrated mobile platforms relies heavily on the sales and marketing functionality of their respective app stores. The number of health and wellness apps available in the app store has been estimated to be 350,000 apps worldwide with as many as 90,000 new health apps added in 2020 alone^2^. The Apple App Store first opened in 2008 and the Google Play Store in 2012, with an emphasis on games, utility apps, and social networks. Barriers to submitting an app were originally low, which meant amateurs or small developer groups could easily make apps. In the health domain this quickly became problematic, as a 2015 review of asthma apps and insulin calculators found, which identified a range of potentially harmful errors and privacy issues^3–5^. Today, Apple and Google selectively assess apps with a health impact. For example, in 2019 apps associated with e-cigarettes or “vapes” were removed and in 2021 during the height of the COVID-19 pandemic the app of an online dating community for people who chose not to be vaccinated against the coronavirus was removed^6^. Both companies also sell a range of products with health implications such as the Apple Watch, which in its latest version includes a heart rate sensor, an electrocardiogram, and an irregular heart rhythm notification. The Apple App Store currently features a category of “Health and Fitness” covering a range from simple wellness apps that play white noise or sleep diaries all the way up to more clinically relevant apps such as a self-harm tracker, heart rate monitors, and mental health counselling platforms. At the time of writing, apps in this category do not carry details of any medical device certification. Some top-rated apps in this category simultaneously make claims like “instantly measure heart rate” or “accurate like ECG” but disclaim “not intended for medical use, for fitness use only”. A 2020 assessment of the regulatory status of paediatric drug calculator apps made available to the EU market, which have an intended purpose classifying them as medical devices, found that one only out of 74 apps (1.4%) had the necessary regulatory approval^7^. Of these apps, 66 of the 74 (89.2%) apps were available on Google Play Store and 8 (10.8%) on Apple App Store.

**Fig. 1:**
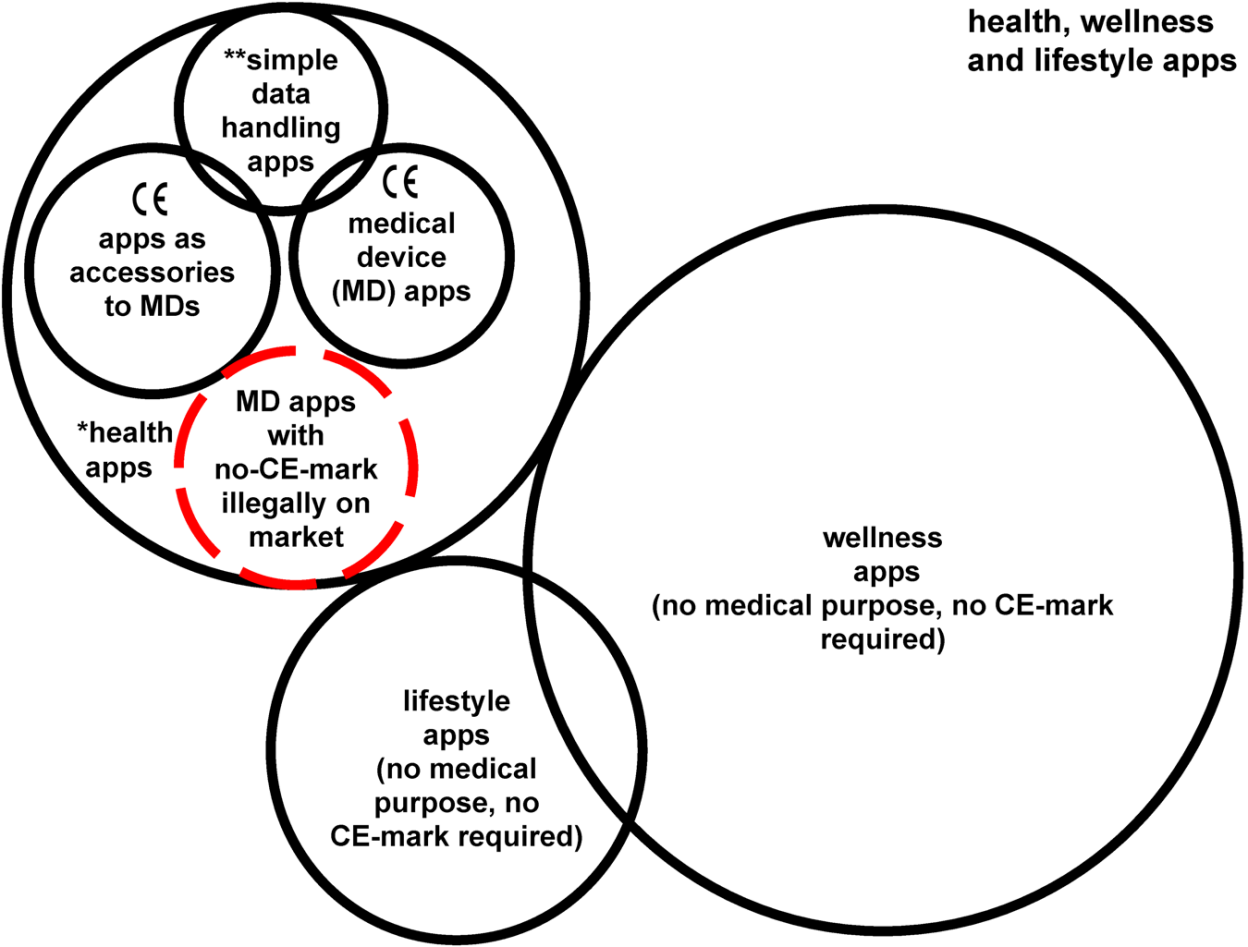
Categories of apps in the health, wellness, and lifestyle area. *MD* Medical Devices, *CE* Conformité Européenne. *health apps: this group includes apps that must be CE-marked, and simple data handling apps (**) for the transfer, communication, compression, storage, conversion, formatting, archive, display or simple search of medical information, which must only be CE-marked if they have overlapping MD functionalities.

Increasingly, government agencies such as the US Food and Drug Administration (FDA)^8^, UK’s Medicines and Healthcare products Regulatory Agency (MHRA)^9^, Germany’s Bundesinstitut für Arzneimittel und Medizinprodukte (BfArM, or the Federal Institute for Drugs and Medical Devices) and the EU Medical Devices Coordination Group (MDCG) have delineated where a “health app” crosses into being a “medical device”^10,11^. While there is local variation, broadly speaking a “medical intended purpose” includes prevention, diagnosis, monitoring, or treatment of a disease, injury, or handicap^12^. Software with a general purpose used in a healthcare setting (such as a word processor or spreadsheet), that is a simple fitness monitor, provides education, or fulfils back-office functions like booking appointments are excluded. However, the proportion of health and wellness apps that are (or should be) registered as medical devices in various regions is not readily discernible from public app store data. Apps which have been developed for a medical purpose must be marked to indicate that they are in conformity with applicable regulations and quality management standards (Conformité Européenne, “CE-marked”) before being made available in the 27 countries of the European Union (EU), the EEA member states that implement EU medical devices regulation (Iceland, Liechtenstein, and Norway) and Turkey (together the “Union” for the purpose of the EU Medical Devices Regulation (EU) ‘MDR’ 745/2017) (Fig. 1)^12–14^. In accordance with the EU MDR, which replaced the EU Medical Devices Directive (‘MDD’ 93/42/EEC), manufacturers are required to label their products with the CE mark to signify they comply with specific standards of performance, quality, safety, and efficacy for their product type. Depending on the nature of the product, a range of quality management system (QMS) procedures must be put in place such as ISO13485, with a range of documents produced that must then be audited, in most cases, by a “notified body”. Over the past five years the EU has upgraded the regulatory risk classification of many ‘Software as a Medical Device’ (SaMD) apps, and hence the oversight processes required (see Table 1), through EU MDR which became applicable on 26 May 2021^15^. Apps which perform simple data handling tasks, including the transfer, communication, compression, storage, conversion, formatting, archive, display or simple search of medical information are not included in this definition^11^. MD apps providing information to be used to take decisions with diagnostic or therapeutic purposes are classified moderate risk (i.e., class ‘*IIa*’, or in higher risk classes (‘*IIb*’ or ‘III’) if the decisions made on the basis of their information have a serious (serious health deterioration or the need for surgery) or a critical impact (death/irreversible health deterioration)^12^. Medical device apps include those supporting healthcare delivery through advice to users on their symptoms, apps interacting with wearable or smartphone/ smartwatch sensors, and digital therapeutics.

**Table 1:** New duties for the app stores in the EU as a result of MDR replacing MDD for medical device apps.

Responsibility of an app store as distributor of medical device apps	Prior to EU MDR^12^, under EU MDD (93/42/EEC)^29^.	New controls under EU MDR (EU 2017/745) from May 2021^13^.
Register on the EU database EUDAMED	✘	If required by Member States, and mandatory once EUDAMED is fully functional
Maintain a register of complaints	✘	**✓**
Cooperate and comply with requests of regulatory authorities	✘	**✓**
Reporting to regulatory authorities of the Member State in which you are registered	✘	**✓**
Requirements for detailed verification of product descriptions and labelling	✘	**✓**

App stores must ensure that apps meet EU safety and health requirements when acting as a distributor. If the app store is also acting as the importer, they are subject to broader controls, including their own registration on the EU database EUDAMED^18^, and confirmation that the MD app is registered on the database, as described in^13^. Current requirements relate to medical device apps only, but EU legislation under consideration may add additional requirements for wellness apps^22^.

### App store medical device oversight

**Fig. 2:**
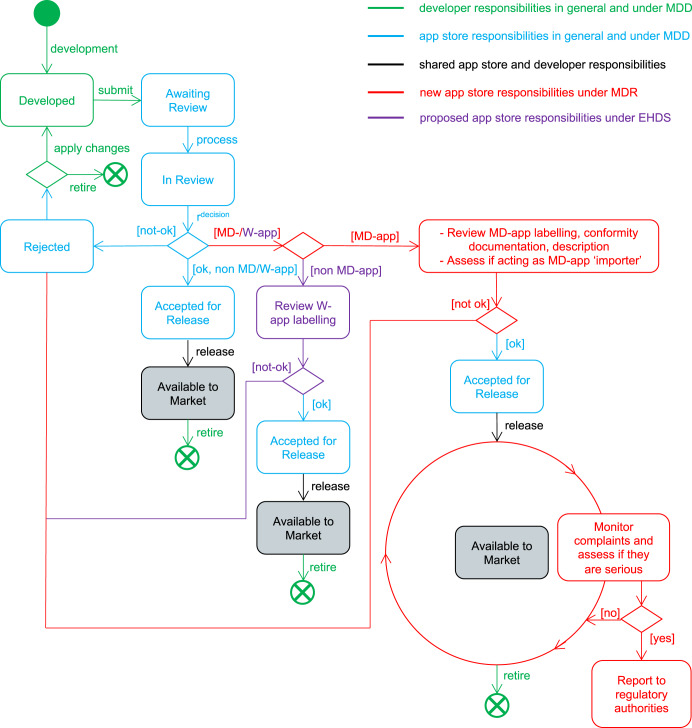
App stores approval processes. Historical, new, and proposed app store approval requirements in the EU, described in the context of general apps, wellness apps (W-apps), and medical device-apps (MD-apps). This is based on processes used by the Apple App Store, as reported in^28^. Currently similar requirements do not extend to W-apps, but this is under consideration as part of the European Health Data Space (EHDS) proposal.

Prior to the application of the EU MDR, there were no specific requirements for importers or distributors. As such, app stores and particularly app stores which ‘imported’ apps from outside the EU had limited legal responsibilities, and regulatory authorities had limited means of enforcement. Since the new regulation came into force competent authorities have greater powers with respect to importers or distributors, but it remains unclear the extent to which the app stores have adapted to their new roles set out in Table 1 and Fig. 2. App developers are responsible to identify if their apps quality as MD apps and to comply with the rules for app development, approval and placing on the market. They must develop apps according to MDR requirements if the MD-apps are to be made available in the EU^12^. Under MDR, the app store is also responsible to ensure they only distribute and import compliant MD-apps, through strict approval verification processes, and that they monitor for and report serious complaints^12,13^. App stores have a number of specific requirements they impose on developers submitting medical device apps or wellness apps to them, that are in addition to the requirements for non-health related apps (Table 2).

**Table 2:** App store requirements for developers of health apps that do not apply to non-health applications.

Requirement	Apple App Store^30^	Google Play Store^31^
Market authorisation document required alongside submission to app store for approval	**✓**	✘ Not requested- there is a general requirement that apps are compliant with applicable laws and regulations.
Manufacturer must disclose, with their submission, the data and methodology used to support the accuracy of claims that relate to health measurements	**✓** Apple states that they will reject apps for which they cannot validate the level of accuracy, or the methodology used to support these claims.	✘ Not requested. There is a statement that apps must not have misleading health claims, that contradict existing medical consensus, or can cause harm to users.
Functionality in the app to remind users to consult with a doctor in addition to their use of the app before they make medical decisions.^a,b^	**✓**	✘
Additional requirements on the types of organisations who can make drug dosage calculator apps available, verified at the time of submission.^b^	**✓**	✘
Apps that make unsupported claims (in the app or in the app store metadata) regarding physiological measurements without dedicated sensors for claimed medical purposes will not be approved.^c^	**✓** Excludes functionalities of measurement through inbuilt smartphone sensors, such as measuring blood pressure, body temperature, blood glucose levels, blood oxygen levels or internal imaging.	**✓** Requires: (i) this use case be only provided through interaction with dedicated and specifically designed smartphone sensors; (ii) the apps must contain disclaimers in the app store metadata stating that they are not intended for medical use and that they are only designed for general fitness and wellness purposes and are not a medical device; and, (iii) must properly disclose the compatible hardware model/device model.

^a,b^ these are not specific EU requirements for app stores; ^c^ This app functionality is a focus area of US FDA enforcement activity^16^.

Overall, the approach of the app stores to the approval and release of apps has remained largely unchanged. Their requirements on developers have been updated to minor degrees to address specific concerns regarding sensor-based health data measurement, possibly in reaction to the US FDA’s focus on this topic^16^. EU MDR, applicable since May 2021, goes further than either Apple or Google’s current approaches^12^. The EU trade body for software medical device manufacturers, COCIR, interprets the role of the app stores to be “distributors”^13^. “Distributors” are defined as those actors in the delivery chain, other than importers and developers, who make a device available on the market. If a MD app developer not based in the Union, makes its app available in the EU via an app store, then the app store is not only the distributor of the MD app, but also its “importer”. Most, but not all the requirements on distributors and importers are already met by Apple, but as of yet, Google meets only a small subset of these requirements (Tables 1 and 2). App stores must ensure through verification checks that apps comply with specific requirements in the regulation EU MDR (as described in Table 2, and in^13^), and remove noncompliant apps. As distributors, they are permitted to do this on a sampling basis rather than having to audit every app. Where apps rely on software that is run on infrastructure outside the Union for its functioning (e.g., an AI model that provides information for diagnostic or therapeutic purposes shown in the app as output) this software should comply with the CE marking requirements of EU MDR, based on Article 6 of that Regulation.

The traditional pre-digital role of distributors was to purchase products from manufacturers to market and sell these products to end-customers. This would typically involve coordination of storage and transport, however, activities such as complaint management was not specifically required. In the era of cloud-based app distribution, app stores manage information which is critical to safety, and are the principal actors responsible for the handling of complaints. The legislative requirements on app store manufacturers introduced by the MDR include the monitoring and reporting of safety-relevant complaints in app reviews or discussion forums as user feedback (even star ratings), which could contain safety-related information^13^. In addition to checking CE-marking status, instructions for use and labelling, distributors must ensure that devices have appropriately applied unique device identifiers (UDIs) for version tracking, as well as informing the authorities if they become aware of serious issues associated with CE-marked apps. However, there is no evidence in the literature, or in online complaints databases, to show that app stores have ever reported any issues originating from users to regulatory authorities. A search of the US FDA manufacturer and user facility device experience (MAUDE) database reveals no reported complaints about apps distributed through an app store^17^. In the EU, it is unknown, if complaints will be recorded by app stores via the European Database on Medical Devices (EUDAMED), once it is fully operational^18^.

In addition to being distributors, if a medical device app was developed outside the EU, app stores are also effectively functioning as “importers”, which invokes substantial further requirements^13^. Importers must be established in the EU, and while Apple and Google do have subsidiary entities, their role with respect to the EU MDR is unclear. Importers must also ensure that instructions for use are made available and that apps are correctly registered in EUDAMED^18^. Importers of medical device apps must themselves be registered, for each app they import, in this database, or a national equivalent, as long as EUDAMED is not yet functional. At the time of writing (December 2022) Google is not yet registered in this database and Apple is only registered as an importer under Apple Distribution International Limited (Ireland), for Apple Inc. (US)’s own manufactured medical devices and apps. The database is mandatory in some (but not all) EU countries, as it is not yet fully functional at the time of writing. It may be that the app stores have not yet acted as importer to a country for which registration is compulsory, but this is unlikely. Alternatively, the app stores may have registered locally, pending EUDAMED becoming fully applicable throughout the Union.

### Fairness and competition in digital markets

At the same time as app stores are facing increasing responsibilities for the compliance and quality oversight for medical device apps they distribute, the companies behind them are also increasingly developing their own medical device apps. For example, Google has developed a CE-marked app, DermAssist, that helps consumers find personalised information about their skin concerns, including through using the smartphone camera^19^. This creates a potential conflict of interest for Apple and Google, even if the responsibilities for app development and app stores are carried out by different departments and divisions. Independent app developers have no choice other than to use distributors who are also two of their chief competitors. As they already have scale and platform advantage, they can control the order in which apps are presented or highlighted in the app store or can even pre-load their software on hardware devices through their operating system. Their distributors and competitors are therefore in a position of monitoring compliance and carrying out enforcement. It also creates dilemmas for Google and Apple. Independent app developers are likely to perceive bias when their apps are refused access to the app stores, based on perceived unfair assessment of the completeness of compliance aspects of submissions and of supporting validation data. This perception of bias is particularly marked where Apple or Google already have a competitive app in the store, or if they are perceived to be developing or partnering in the development of similar apps. As the activities, investing, and partnering of Google and Apple in health are so broad, conflicts of interest could be perceived in almost any medical application area or app intended purpose.

These dilemmas are relevant in the light of two closely linked recent EU laws, the Digital Markets Act (enforced from November 1st 2022) and the Digital Services Act (enforced from November 15th 2022)^20,21^. The Digital Markets Act recognizes that large online platforms act as “gatekeepers” in digital markets and “have the capacity to affect a large number of end users and businesses, which entails a risk of unfair business practices”. Specifically, businesses who depend on the gatekeepers will have a legally enforced fair business environment, which must ensure that innovators and technology start-ups can compete (i.e., have fair access to the online platform environment) without having to comply with unfair terms and conditions limiting their development. The act provides for the dynamic update of obligations for gatekeepers with remedies to tackle systematic infringements, and harsh penalties of up to 20% of worldwide revenue for repeat infringement, and the ability to insist on divestiture of parts of a business in case of systematic infringements. The Digital Services Act introduces enhanced supervision of platforms, and requirements for platforms to provide better mechanisms for users to flag issues with products or services. Fines for infringement can range between 1–6% of total turnover in the preceding financial year.

**Fig. 3:**
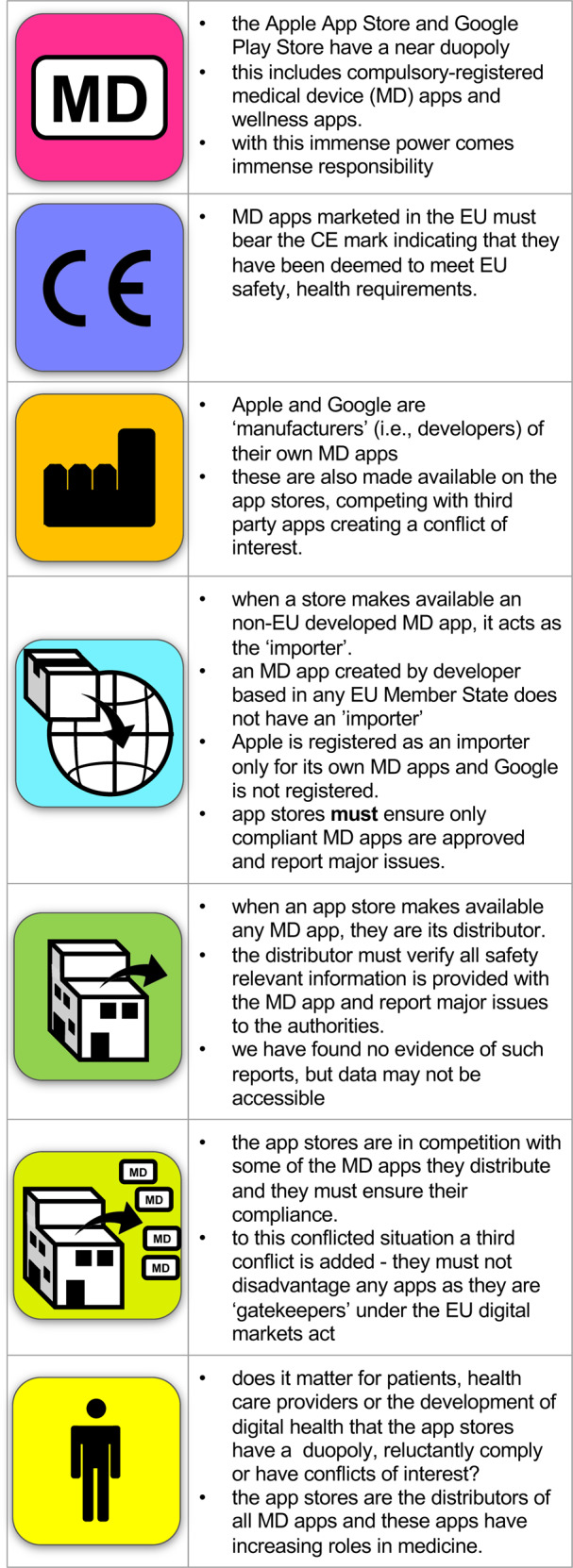
The roles of app stores in compliance. Overview of the challenges for app stores in balancing their medical device compliance role, their role as MD app developers and their obligations as gatekeepers in the EU digital market. Similar app store responsibilities for wellness apps may be introduced under the proposed legislation. *MD* Medical Devices, *CE* Conformité Européenne.

It is quite likely that independent app manufacturers will launch legal claims to pursue app stores on the basis of unfair market practices under the Digital Markets Act for having exercised unfair judgement as both gatekeepers and competitors (Fig. 3).

### App store oversight of wellness apps

Another upcoming legislative initiative in the EU ‘Digital Decade’ program is the European Health Data Space, which is anticipated to become applicable from 2025^22^. It includes provisions for the regulation of wellness apps and has the aim of supporting individuals in taking control of their health data and to increase the use and sharing of health data for better healthcare delivery and health research. The legislation seeks to introduce a voluntary labelling scheme for wellness apps which are interoperable with electronic health record systems (EHRs)^22^. Furthermore, the legislation proposes an EU database where certified EHR systems and labelled wellness applications will be registered. Wellness apps are defined as those used for ‘*processing electronic health data for other purposes than healthcare, such as well-being and pursuing healthy life-styles*’^22^. Although the regulation would predominantly apply to the developers of wellness application and the EHRs with which they interact, it also sets out requirements for the distributors and importers of wellness applications. If labelling is applied by the app developer, it must meet the requirements specified in the act and associated standards. The distributors of the apps, envisaged to include the app stores, must in turn make the label available to customers^22^. As wellness apps are more numerous, the workload for oversight may be greater than for medical device apps, even though there are fewer provisions for distribution.

### EU health and wellness app stores of the future

One vision of the future is that there will be no change in structure or behaviour of the status quo duopoly. The degree to which Apple and Google will experience the enforcement of the provisions of the medical device, the European Health Data Space Regulation, the Digital Markets Act, the Digital Services Act, and the proposed AI Act remains to be seen. In the past large platform operators expected a degree of “digital exceptionalism”—the view that digital approaches and digital medicine are different and that special rules apply, particularly for the large US-based platforms^23^. The principles behind the centrepieces of the new European digital strategy are that they apply to all, and that there are specific requirements and tough penalties, particularly for gatekeeper platforms.

An alternative vision of the future is created by a provision of the digital markets act that could force Google or Apple to allow competing app stores for their iOS and Android environments. The implications of this are lesser for Google, as alternative Android app stores do exist, e.g. for free/open source apps only^24^. By contrast, Apple has strongly resisted ‘sideloading’, i.e., the opening up of iOS to apps not provided from the Apple App Store^25^, and has only since the enforcement of the Digital Markets Act considered this^26,27^. In the health app sector, specialised and regulated digital app ‘pharmacies’ could be developed that run as independent organizations providing curation, oversight, triage, documentation, and complaint handling for apps. Such organizations could be run as for-profit entities (as are brick and mortar pharmacies in the real world), non-profits funded by grants, or national governments. Unlike the existing app stores, digital app pharmacies would have no fundamental conflicts of interest with developers. These app stores would still incur the regulatory oversight costs associated with being a medical device distributor or importer, but could recoup costs through registration fees to developers, fees charged on in-app payments, or direct fees to consumers.

A third vision of the future of EU health and wellness app stores is that the current duopoly remains, but that Google and Apple evolve their approach substantially to resolve the developing pressures and meet legislative requirements fully and transparently. This would involve developing approaches to fully deliver their responsibilities as distributors and/or importers of medical device apps. This would increase the requirements applied to app developers at the point of submission and update to the app stores, with increased checks carried to verify regulatory conformity and assess data validating claims. This would require active surveillance of complaints and reviews of data by the app stores, alongside reporting of adverse or serious adverse events, as reportable by law under the MDR. The fulfilment of responsibilities without unfair market practices and with the avoidance of public confusion would be more likely if there was separation of app stores into clearly demarcated domains which provide: (i) prescription-only medical device apps; (ii) other medical device apps; (iii) labelled and conforming wellness apps; and, (iv) other non-regulated lifestyle applications. These approaches would need to be accompanied either by divestiture of Google’s and Apple’s own medical device app development businesses, or the thorough separation of these businesses from their app stores business. The separation must be sufficient to convince app developers, EU regulators and courts that conflict of interest was adequately managed. Medical devices and wellness apps are already important to consumers, to patients and to health systems. To enable this sector to further develop safely, these ‘wild west’ aspects of the market must be resolved.

### Reporting summary

Further information on research design is available in the Nature Research Reporting Summary linked to this article.

## Supplementary information

Reporting Summary

## Data Availability

Not applicable
